# Chats at St. Ampelio
*"Chats at St. Ampelio." By John A. Goodchild. (Kegan Paul, Trench and Co.)


**Published:** 1890-02-08

**Authors:** 


**Chats at St. Ampelio.***

Mankind owes much more gratitude than it usually expresses to those who have written books that serve to occupy the odd minutes of life. The great authors demand our leisure hours; we take them a canto at a time. The minor poets and essayists charm the spare five minutes. We may read a stanza or two of Herrick and Béranger, or a chapter of Montaigne, and put the book down without feeling that we have done the author an injustice. Such gratitude is due to Dr. Goodchild for his "Chats at St. Ampelio." From dedication to *finis* his book is entirely charming, freshly written, full of quaint conceits and dainty suggestions. His humour is delightfully spontaneous and refreshing, and while he may be held quite innocent of plagiarism, there are many touches which recall blended recollections of Wilson, Lamb, and Helps. This is high praise, but we think well merited. It is impossible to select quotations at all representative of the tone of the book, but perhaps the chapter on "Sorrows and Symbols" is one of the most delightful. This is one of those books that we not only advise people to read, but to possess, and it will serve to while away, with pleasure and profit, many a spare quarter of an hour, and many a sleepless night.

